# Impact of Branched Chain Amino Acid on Muscle Mass, Muscle Strength, Physical Performance, Combined Survival, and Maintenance of Liver Function Changes in Laboratory and Prognostic Markers on Sarcopenic Patients With Liver Cirrhosis (BCAAS Study): A Randomized Clinical Trial

**DOI:** 10.3389/fnut.2021.715795

**Published:** 2021-09-22

**Authors:** Arun Singh Tejavath, Amit Mathur, Deepak Nathiya, Pratima Singh, Preeti Raj, Supriya Suman, Payal Ramakant Mundada, Sheikh Atif, Ramesh Roop Rai, Balvir Singh Tomar

**Affiliations:** ^1^Department of Gastroenterology, National Institute of Medical Sciences, Nims University Rajasthan, Jaipur, India; ^2^Institute of Pharmacy, Nims University Rajasthan, Jaipur, India; ^3^Institute of Pediatric Gastroenterology and Hepatology, Nims University Rajasthan, Jaipur, India

**Keywords:** sarcopenia, liver cirrhosis, branched-chain amino acid, albumin, quality of life

## Abstract

**Background:** This study aimed to investigate the long-term effects of branched-chain amino acids (BCAAs) supplementations on the parameters associated with improved prognosis in sarcopenic patients with liver cirrhosis (LC) and evaluate its impact on cirrhotic-related events.

**Methods:** A 24-week, single-center, randomized, open-label, controlled, two cohort parallel-group intervention study was carried out by comparing the efficacy of BCAAs against lactoalbumin (L-ALB) on 106 sarcopenic patients with LC. The BCAA (intervention) group was treated with 7.2 g BCAA per dose, whereas the L-ALB group was treated with 6.3 g of L-ALB. The primary outcome was to assess the effect of BCAA on the parameters of sarcopenia, such as muscle mass, muscle strength, and physical performance. The secondary outcomes were to study the combined survival and maintenance of liver function changes in laboratory and prognostic markers over the duration of 6 months.

**Results:** The treatment with BCAA leads to the significant improvement in sarcopenic parameters, such as muscle strength, muscle function, and muscle mass. The total cirrhotic-related complications and cumulative event-free survival occurred fewer in the BCAA group than in the L-ALB group. In addition, prognostic markers improved significantly in the study.

**Conclusion:** The current study demonstrated that long-term BCAAs supplementation improved sarcopenia and prognostic markers in patients with advanced LC.

## Introduction

The liver is a prime site for biochemical pathways responsible for nutrient metabolism. Cirrhosis-related liver dysfunction results in nutritional diseases, such as sarcopenia, which indicates severe muscle protein depletion (1, 2). Furthermore, sarcopenia is a syndrome characterized by progressive and generalized loss of muscle mass, which also accounts as a major predictor for morbidity and mortality in people with liver cirrhosis (LC) (3). Indeed, sarcopenia is one of the most commonly prevalent metabolic complication which is associated with reduced levels of branched-chain amino acids (BCAAs) (3–5). Patients with LC are in the hypermetabolic state as their energy generation pattern after an overnight fast is parallel to that observed in healthy individuals after 2–3 days of starvation (5, 6). Such a catabolic state increases the depletion of amino acids and accelerates the breakdown of skeletal muscle to release amino acids with disruption of muscle balance, resulting in sarcopenia (7–9). The poor prognosis of sarcopenia adversely affects the survival, health-related quality of life, and response to any underlying infection.

Recent studies conclude that BCAAs preparations have been proposed to be effective in improving the body composition by correcting the amino acid imbalance in the blood in patients with decompensated cirrhosis (10). However, most of the studies are conducted on a small sample size with multiple parameters, such as different complications of LC (11). Hence, there is an utmost need for a longitudinal study to assess the impact of BCAA, as the Indian population is experiencing a temporal trend on patients with sarcopenic cirrhosis (12).

In this regard, we report the long-term benefits of BCAA on the parameters associated with improved prognosis in sarcopenic patients with LC. Additionally, we have evaluated its impact on the cirrhotic-related events.

## Methods

### Study Design and Setting

A 24-week, single-center, randomized, open-label, controlled, two cohort parallel-group intervention study on the LC was carried out in the Department of Gastroenterology, National Institute of Medical Sciences and Research, NIMS University Rajasthan, Jaipur, India, from April 2019 to October 2020. The protocol was conformed in accordance with the 1,975 principles of the Declaration of Helsinki and was approved by the Institutional Ethics Committee (IEC No NIMSUNI/IEC/217/22) (3) and was registered under Clinical Trials Registry—India (CTRI/2020/04/024762). Each participant was informed in detail about the purpose of the study, and the treatment was fully explained to all participants before obtaining informed consent. The manuscript conformed to the Consolidated Standards of Reporting Trials Statement 2010 (CONSORT 2010 Statement) to improve the quality of reporting.

### Participants

In this study, the population consisted of patients with cirrhosis who met the inclusion and exclusion criteria. The inclusion criteria were: diagnosis of LC based on the clinical and laboratory data and/or liver biopsy specimens, Child-Turcotte-Pugh Score (CTP) ≥ 7 and < 12 (class B or C), radiological and endoscopic evidence of portal hypertension with sarcopenia, according to the cut-off values of European Working Group on Sarcopenia in Older People 2, 2018 (EWGSOP2) (13), the ability to stand with or without support, receipt of adequate nutrition (sufficient energy intake according to the requirements set by a registered dietitian), and provision of informed consent. The exclusion criteria were as followed: active alcohol intake (in the past 6 weeks), active malignant disease, overt hepatic encephalopathy, severe/refractory ascites, CTP score ≥ 12, active gastrointestinal bleeding, renal failure (serum creatinine> 1.5 mg/dl), brittle diabetes mellitus, psychiatric/neurological problems, hepatocellular carcinoma, history of a previous transjugular intrahepatic portosystemic shunt, marked symptomatic comorbidities (cardiac, pulmonary, and renal), and non-compliance with treatment.

### Intervention Protocol

Patients after fulfilling the baseline evaluation were randomized and sub-classified into two groups, lactoalbumin (L-ABL) group and BCAA group, for supplement provision. Both the sachets (active and placebo) weighed equally (10 g). Explicitly, BCAA (active treatment) weighed 8.1 g containing 1.2 g L-leucine, 0.6 g L-isoleucine, and 0.6 g L-valine and saccharose (5.7 g) for a total energy supply of 37.5 kcal. The L-ABL group weighed 9.1 g, which consisted of 2.1 g L-ALB (90.3 mg valine, 226.8 mg of leucine, and 126 mg iso-leucine) with 4.0 g saccharose, and 3.0 g mannitol for a total of 33.6 kcal/packet (supplied by—Medisys Biotech Pvt Ltd, Sirmour, Himachal Pradesh, India) (11). The patients were randomized into the BCAA and L-ALB group in a 1:1 ratio using a simple randomization method (Microsoft Excel 2010; Microsoft Corporation, Washington DC, WA, USA). During the study period, the total energy and protein were kept at 30–35 kcal kg^−1^ d^−1^ and 1.5 g protein kg^−1^ d^−1^ through the provided supplement. With all, the nutritional supplementations were closely equicaloric and equi-nitrogenous, according to the 57th recommendation of European Society of Parenteral and Enteral Nutrition guidelines (14). During the whole experimental period, participants recorded their nutritional supplement intake timing and amount in the paper log provided by the investigators. The compliance to the treatment was assessed after counting the number of packets left in the original packet by the investigator in the follow-up, ≥ 90% adherence was kept adequate to continue with the eligibility.

### Outcomes

The primary outcomes of the study were to assess the impact of BCAA on the parameters of sarcopenia, such as muscle mass, muscle strength, and physical performance. The secondary outcomes were to study the combined survival and maintenance of liver function, weighed through death or deterioration to exclusion criteria with changes in the clinical laboratory, and prognostic markers in the duration of 6 months.

### Baseline and Follow-Up Clinical Assessment and Investigations

An overview of data collection (demographic and clinical) characteristics of baseline allocation in both the groups is given in Table 1. The patients were instructed to visit the department of gastroenterology for a consecutive period for further follow-up. After confirmation of diagnosis, all the assessments were conducted during the enrollment considered as a baseline followed by the 12 and 24th week.

**Table 1:** Baseline clinical characteristics of 138 patients enrolled in the study.

**Characteristics**	**BCAA group (*N* = 69)**	**L-ALB group (*N* = 69)**
Age (year)	43.69 ± 15.50	48.02 ± 13.02
Male	60 (86.95)	58 (84.05)
Weight (Kg)	55.04 ± 6.27	55.40 ± 9.84
Body Mass Index (Kg/m^2^)	21.06 ± 2.65	22.09 ± 3.54
Etiology (Alcohol/ Viral/ Autoimmune/ Cryptogenic)	51/11/1/6	46/13/4/6
Ascites (Mild/Moderate/Severe)	11/35/11	12/15/24
Child-Turcotte-Pugh Score	10.22 ±1.44	10.74 ± 1.43
Child-Turcotte-Pugh Class B/C	33/36	34/35
MELD	13.89 ± 2.31	13.81 ± 3.05
Hepatic Encephalopathy (None/Grade I/Grade II)	48/12/9	46/13/10
ALT	30.0 (18–40)	29 (17–41)
AST	68 (33–112)	65 (32–109
Ammonia	71 (40–122)	67 (51–116)
INR	1.60 (1.30–1.86)	1.51 (1.26–1.91)

*Values are expressed in mean and SD (Mean ±SD), number and percentage n (%), and median and interquartile range (IQR).*

*MELD, Model for End-Stage Liver Disease; ALT, alanine transaminase; AST, aspartate aminotransferase; INR, international normalized ratio*.

#### Anthropometric Assessments

The subjects were weighed and measured for height to calculate the body mass index (BMI = actual weight [kilogram]/ height [meter square]). The triceps skinfold (TSF) was measured in millimeters using a skinfold caliper (Harpenden–weight (with case): 3 pounds (1.8 kg); Dial Graduation: 0.20 mm; measuring range: 0–80 mm; measuring pressure: 10 g mm^−2^ (constant over range); accuracy: 99%; repeatability: 0.20 mm). Mid-upper arm circumference (MUAC) was measured in centimeters, using soft fiberglass tape (Foshan Guo's Wintape Measuring Tape Co., Ltd., Guangdong, China Length: 762 mm Width: 2.5 mm), which is wrapped around the mid-upper arm at the mid-point between the olecranon and the acromial process. After obtaining the values of TSF and MUAC, mid-arm muscular circumference (MAMC) was calculated using the formula: MUAC (centimeters) – [TSF (millimeters) × 0.314]. The average of three consecutive measurements was then recorded and included in the analysis. An anthropometric evaluation was performed according to the manual of the International Society for the Advancement of Kinanthropometry (ISAK) return (15). The values obtained were compared with the reference values of the National Health and Nutrition Examination Survey (NHANES) on Frisancho tables to classify significant malnutrition status in the enrolled patients (16, 17).

The muscle strength was assessed by hand grip strength (HGS) using a hand dynamometer [Takei TKK 5401 Digital Handgrip Dynamometer, Takei, Niigata-City, Japan, measuring range: 5.0–100 kgf, accuracy: ± 2.0 kgf, dimensions: ~154 (W) × 235 (D) × 62 (H)mm, and weight: ~0.63 kg]. HGS was expressed in kilograms with two decimals. Measuring grip strength is a powerful and simple measure to assess muscle strength (18, 19). The average of three consecutive measurements was then recorded and included in the analysis. Gait speed was assessed using a modified 6-m walk test in a standardized manner to assess the physical performance (20, 21). Low grip strength corresponds to cut-off points <26 kg m^−2^ for men and < 18 kg m^−2^ while low gait speed was predicted with < 0.8 m s^−1^. The muscle quantity was measured using MRI, which is considered a gold-standard, non-invasive method for muscle mass assessment. MRI is a universally recognized method to quantify detailed tissue structure and composition, facilitating the quantification of muscle volume. The area was calculated using MRI imaging by a trained operator, with image AsanJ-Morphometry software (Asan Image Metrics, Seoul, Korea) that was developed for abdominal muscle and fat area measurements based on ImageJ (NIH, Bethesda, MD, USA). Total abdominal muscle area (TAMA) was measured at the L3 vertebral level for the diagnosis of low muscle mass with sarcopenia specific cut-off (male and female cut-off values for SMI at < 50 and < 39 cm^2^ m^−2^, respectively). Sarcopenia was defined as low muscle strength, low muscle quantity, and low physical performance as per the updated European Working Group on Sarcopenia in Older People 2, 2018 (EWGSOP2) (13).

#### Laboratory and Prognostic Markers Assessment

Laboratory tests, such as serum albumin, serum creatinine, total bilirubin, serum alanine aminotransferase, serum aspartate aminotransferase, and international normalized ratio (INR) were performed. The severity of liver disease was defined using the CTP score and Model for End-Stage Liver Disease (MELD) using laboratory parameters (17, 22).

### Statistical Analysis

The previous study on enriched BCAA supplementation on sarcopenic patients reports 25% improvement in the grip strength (23). According to those estimates, with G-power software, we calculated a sample size of 54 in each group with 80.0% statistical power to observe the effect size 0.5, and two-sided effect in pre-and post-intervention matched pairs with β error of 20%. With the expectation of a 10% drop-out rate due to further deterioration of liver function or development of unpredictable complications, the final sample size was kept at 59 in both the groups.

Baseline characteristics were compared between the two study groups using the Fischer's test for categorical variables and the Mann–Whitney's test for quantitative variables. The changes in the MELD score, CTP score, serum albumin, and bilirubin levels were analyzed using a mixed linear model. The cumulative survival event-free survival (EFS) rates were estimated using the Kaplan–Meier analysis and compared using the log-rank Mantel–Cox test and the Cox-proportional regression model was used to identify the factors that contributed to the prognosis of each patient. The patients were censored when they dropped out of the study (because of hepatocellular carcinoma, death, variceal hemorrhage, or any other reason). Changes in results are expressed in mean ± SD, median (range), or frequencies. *P* < 0.05 was considered statistically significant. Data were analyzed using Statistical Package for the Social Sciences for Windows (software version 25; IBM Corporation, Armonk, NY, USA).

## Results

### Characteristics of Patients and Clinical Course

**Figure 1:**
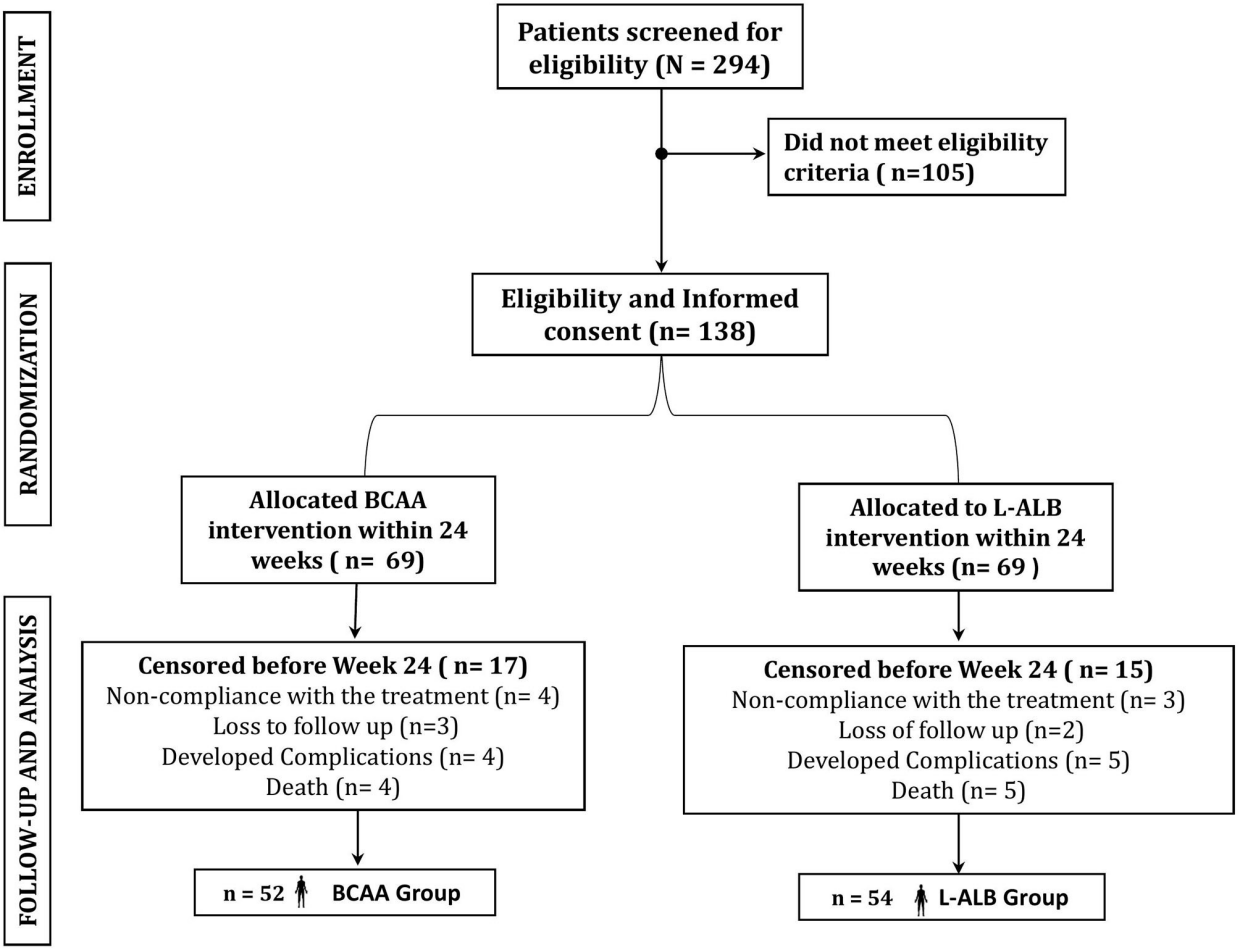
Schematic study plan in consideration of the patients enrolled in the study.

In this study, 294 patients were screened for eligibility as per protocols. Initially, altogether 138 patients were enrolled in the study (Table 1), out of which only 106 patients completed the study with regular follow-up till 24 weeks (52 in the BCAA group and 54 in the L-ALB group) (Table 2). There is no significant difference between the demographic, mean baseline, and clinical characteristics of both arms. The overall completion rate was close to 76.81%, with a drop-out of 23.18%. During the study, 18 patients were censored (non-compliance: seven, loss of follow-up: five, developed complications: nine, and death: nine). Seven patients who withdrew for non-compliance mentioned the poor-palatability of nutritional supplementation as a reason behind the withdrawal (four in the BCAA group vs. three in the L-ALB group). Non-compliance occurred within the first 3 months of the treatment except in the L-ALB arm, in which two patients declined for further treatment after 3 months of the treatment (Figure 1).

**Table 2:** Baseline characteristics of 106 patients who completed the study till 24 weeks.

**Characteristics**	**BCAA group (*N* = 52)**	**L-ALB group (*N* = 54)**	*p*-value^*^
Age (year)	43.05 (15.01)	47.18 (13.07)	0.983
Male	43 (83.69)	44 (81.48)	0.052
Weight (Kg)	55.26 (6.30)	54.24 (7.78)	0.934
Body Mass Index (Kg/m^2^)	21.16 (2.76)	21.53 (3.09)	0.996
Etiology (Alcohol/ Viral/ Autoimmune/ Cryptogenic)	39/8/1/4	36/11/0/7	0.061
Ascites (Mild/Moderate/Severe)	8/23/21	7/24/22	0.052
Hepatic Encephalopathy (None/Grade I/Grade II)	40/8/4	39/10/5	0.049
Child-Turcotte-Pugh Score	9.41 ± 1.52	9.69 ± 1.49	0.540
Child-Turcotte-Pugh Class B/C	33/36	34/28	0.621
MELD	13.64 ± 2.61	13.81 ± 3.13	0.810
ALT	29 (19–40)	29 (18–40)	0.218
AST	68 (33–110)	64 (32–105)	0.079
Ammonia	71 (40–122)	68 (52–118)	0.021
INR	1.62 (1.31–1.89)	1.52 (1.28–1.92)	0.995

*Values are expressed in mean and SD (Mean ± SD), number and percentage n (%), and median and interquartile range (IQR).*

*MELD, Model for End-Stage Liver Disease; ALT, alanine transaminase; AST, aspartate aminotransferase; INR, international normalized ratio.*

* *Statistically significant at p < 0.05*.

#### Outcome of Study

##### Effect on Sarcopenic Parameters (Muscle Mass, Muscle Strength, and Physical Performance)

**Figure 2:**
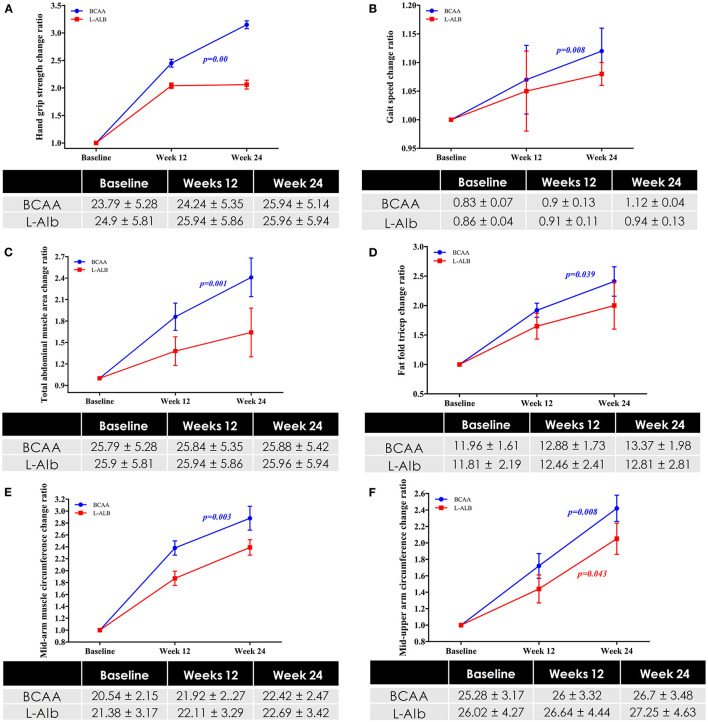
**(A)** Changes in Hand-grip strength in BCAA and L-ALB group over a period of 24 weeks. **(B)** Changes in muscle function in BCAA and L-ALB group over a period of 24 weeks. Changes in muscle mass based on the different variables; **(C)** Total abdominal muscles area (TAMA) circumference; **(D)** Fat-fold triceps; **(E)** Mid-upper arm circumference; **(F)** Mid-arm muscle circumference in BCAA and L-ALB group over 24 weeks. Statistically significant at *p* < 0.05.

The primary outcome of the study was to assess the impact of BCAA on muscle strength, muscle function, and muscle mass in the patients with LC. Concerning muscle strength, the hand-grip strength score was significantly (*p* = 0.00) improved (23.79 ± 5.28 kg) after treatment of 6 months in the BCAA arm (25.94 ± 5.14 kg) (Figure 2A). Furthermore, in terms of muscle function, gait speed score was significantly (*p* = 0.00) improved from before treatment (0.83 ± 0.07 m s^−1^) to after treatment of 6 months (1.12 ± 0.04 m s^−1^) (Figure 2B). For muscle mass, total abdominal muscle area (TAMA), fat fold triceps (FFT), and mid arm muscle circumference (MAMC) were statistically significant (*p* = 0.001), (*p* = 0.039), and (*p* = 0.03), respectively, in the BCAA group with the increased mean of 2.41 ± 0.27, 2.41 ± 0.25, and 2.80 ± 0.20, respectively (Figures 2C–F).

##### Effect on Cirrhotic Complications and Survival

**Figure 3:**
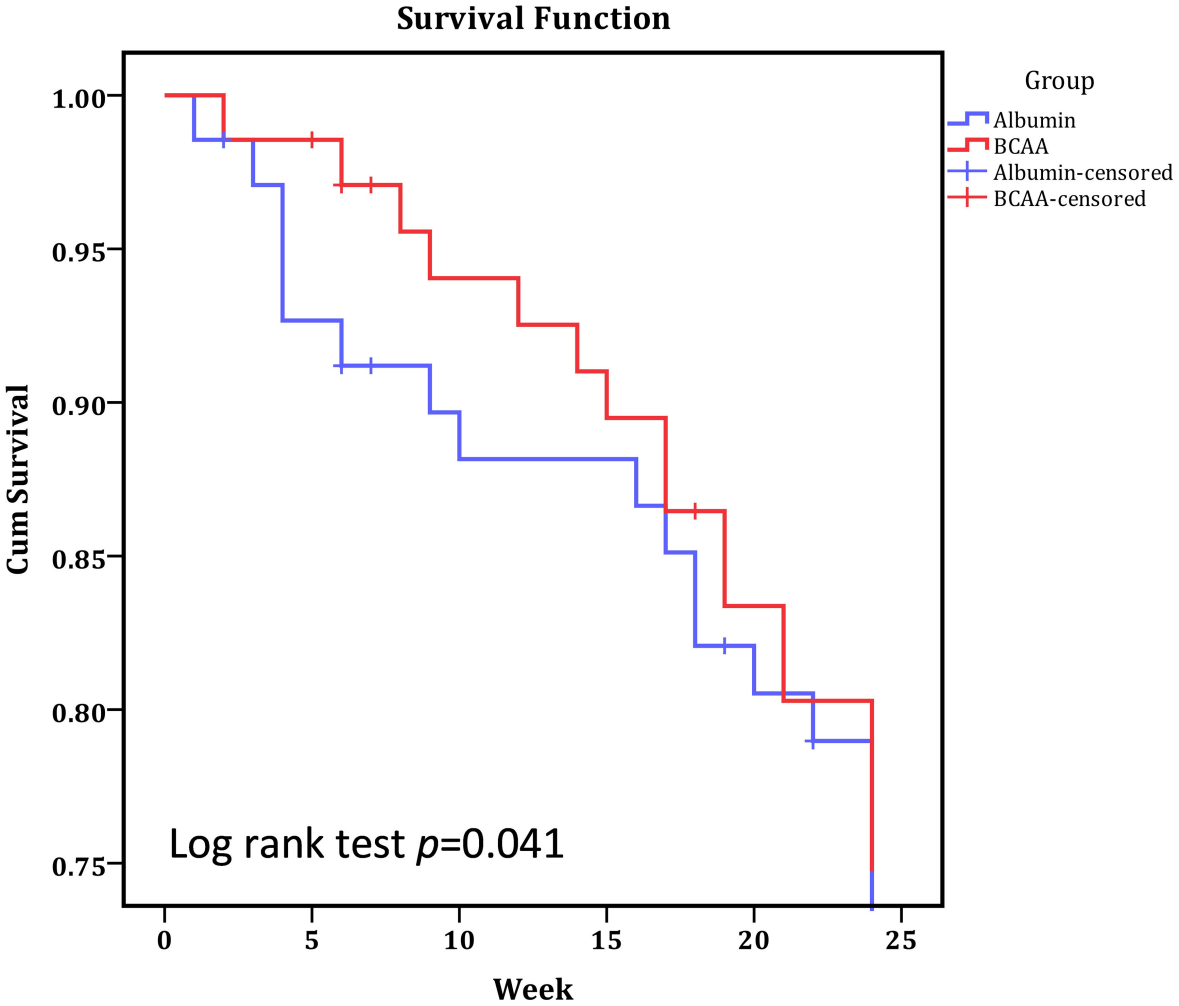
Kaplan Meier analysis of cumulative event-free survival in patients supplemented with BCAA and L-ALB group over 24 weeks. Events were considered death (any reason) and deterioration to exclusion criteria. Statistically significant at *p* < 0.05.

Figure 3 depicts the cumulative event-free survival of the BCAA and ALB groups. There was no significant difference in time course event between the two groups on the basis of intention to treat (log-rank Mantel–Cox; *P* = 0.600). Table 3 shows the incidence rates of major cirrhosis-related events in both the groups. The total cirrhotic-related complications occurred fewer in the BCAA group than in the L-ALB group [*P* = 0.007; odd ratio (OR): 0.823; 95% *CI*: 0.72–1.445]. In comparison to the ALB group, the progression and aggravation of hepatic encephalopathy were significantly lower in the BCAA group. There was no significant inter-category variation in the following complications: variceal bleeding, hepatorenal syndrome, spontaneous bacterial peritonitis, ascites formation, and hepatocellular carcinoma. The cumulative event-free survival was significantly better in the BCAA group 22.31 ± 0.56 weeks (95% *CI* = 21.21–23.61 weeks) than in the ALB-group: 21.11 ± 0.77 weeks (95% *CI* = 19.59 ± 22.63 weeks); *p* = 0.00 (Figure 3).

**Table 3:** Incidence of major cirrhosis related events in BCAA and L-ALB group.

**Events**	**BCAA group**	**L-ALB group**	*p-*value^*^
Number of patients	52	54	-
Loss of follow-up	3 (2.2)	2 (1.4)	0.649
Death	4 (2.9)	5 (3.6)	0.730
Non-compliance	4 (2.9)	3 (2.2)	0.698
Variceal hemorrhage	1 (0.7)	2 (1.4)	0.559
Hepatic Encephalopathy (III Grade)	11 (10.9)	14 (13.0)	0.031
Hepatorenal syndrome	1 (0.7)	1 (0.7)	1.000
Spontaneous bacterial peritonitis	1 (0.7)	2 (1.4)	0.589
Aggravations of ascites	2 (1.4)	3 (2.2)	0.649
Development of hepatocellular carcinoma	1 (0.7)	1 (0.7)	1.000

* *Statistically significant at p < 0.05*.

##### Outcomes on Laboratory Parameters

**Figure 4:**
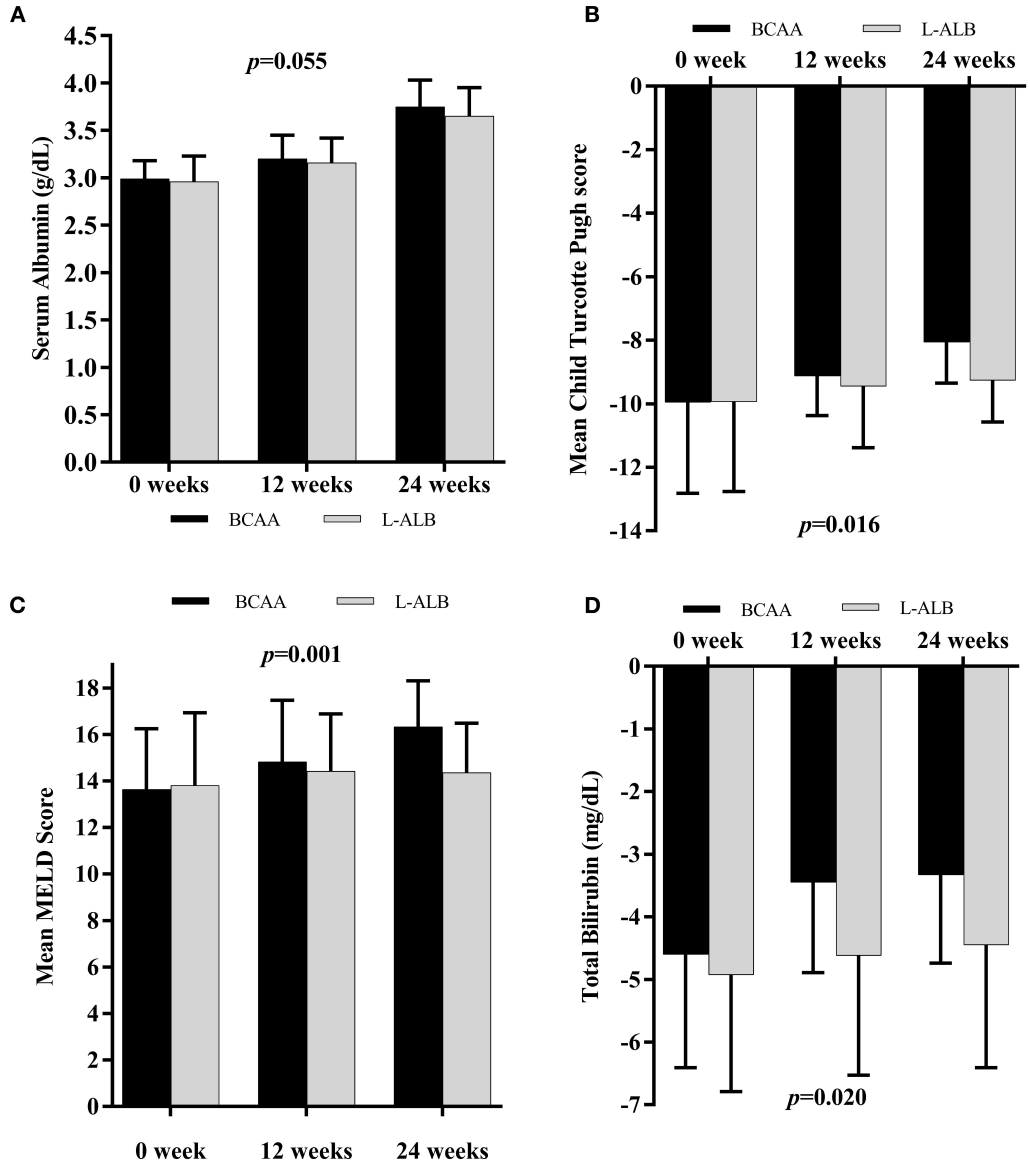
Mean changes in Albumin concentration **(A)**, Child Turcotte Pugh score **(B)**, MELD score **(C)**, and Total Bilirubin level **(D)** in subjects supplemented with BCAA (black scale) or L-ALB (grayscale) over 24 weeks. MELD, Model for End-stage Liver Disease. Statistically significant at *p* < 0.05.

The changes in important laboratory markers over 24 weeks are compared between the two groups in Figure 4. The prevalence and severity CTP score (9.96 ± 2.80–8.06 ± 1.29) and bilirubin level (4.60 ± 1.81–3.33 ± 1.41) decreased and improved significantly in the BCAA group (*p* = 0.020 and *p* = 0.016), respectively (Figures 4A,B). However, there was no significant improvement in the subgroup analysis of serum albumin (Figures 4A,C). The MELD-score decreased significantly (*p* = 0.001) from 13.64 ± 2.61 to 16.34 ± 1.97 in the BCAA group overtime of 24 weeks.

##### Safety Profile and Product Compliance

During the study, the incidence of adverse events and adverse drug reactions were not reported in both the groups (data not shown). The given products were well-complaint among all the patients.

## Discussion

According to the best of our research, there is a scarcity of data on the involvement of BCAA in LC, primarily from the Asian subcontinent. Addressing the dearth of evidence, we account to report the first finding from India. This randomized clinical trial aimed to evaluate the potential benefits of nutritional supplementation with BCAA in decompensated LC. This study results indicated that long-term BCAA supplementation, such as valine, leucine, and isoleucine, has beneficial effects on sarcopenia (muscle mass, muscle strength, and muscle function), with the decrease in the cirrhotic-related complications in several secondary outcomes (CTP score, albumin, and MELD).

In the present study, sarcopenia, as assessed on variables of muscle mass, muscle strength, and muscle function consistent with the updated EWGSOP2, 2018, has improved significantly with BCAA supplementation. One of the key pathophysiologic mechanisms underlying this spectacle is the increase of muscle protein due to mammalian target of rapamycin (mTOR) activation and reduced muscle protein degradation (24, 25). BCAA has a better efficacious energy substrate utilization in contrast to that of glucose and fatty acids. BCAA breakdown primarily takes place in the peripheral tissues (muscles, brain, and adipose tissue), extra-hepatically, so even if the hepatocytes are damaged and incapable of synthesizing protein in the liver, BCAA (especially leucine) in the muscles would promote protein synthesis, as concluded from the previous studies (1, 23, 26). Foremost, cirrhosis-related event, the hepatic encephalopathy, occurred less frequently in the BCAA group than in the ALB group. Early clinical trials have shown the effectiveness of BCAA supplementation on patients with malnutrition and hepatic encephalopathy (27, 28). The metabolism of amino acids is related to the progression of chronic liver disease, resulting in a lower circulating BCAA/aromatic-amino-acid ratio (29, 30). Due to the elevated ammonia levels in the serum and brain, this change can cause hepatic encephalopathy. While there is evidence of the beneficial function of BCAAs in hepatic encephalopathy, there is also contradictory data (30). During oral BCAA supplementation, the EFS improved, such as death from any cause and worsening of liver disease with or without the production of HCC, nonetheless, significant improvement can be better appreciated in a further long-term study (11). The current guidelines of the European Society for Clinical Nutrition and Metabolism recommend taking a BCAA-enriched formula in case of hepatic encephalopathy during enteral nutrition (31).

Other secondary outcomes have improved. The patients supplemented with BCAA exhibited significant improvement in MELD score over time. A Korean, retrospective, observational cohort study showed similar improvement in MELD score, which is a well-known predictive indicator of the pre-transplant waiting list death rate (32–34). However, the advancement of liver disease and progression of MELD score can be slowed by antiviral agents, such as nucleos(t)ide analogs and abstaining alcohol consumption in the existing study. Additionally, nutritional support is known to be an independent factor for improving these outcomes in these patients (32). Interestingly, we found decreased serum bilirubin levels which may potentially be responsible for the lower MELD score in the BCAA group. The results are influenced by the progressive exclusion of subjects who reached the event threshold because ANOVA takes the time course of parameters of subjects that completed the study in all treatment groups. These results are similar to an Italian randomized prospective study that also reports improvement in bilirubin level and CTP scores (11).

Significant related changes in CTP scores were consistent with the Korean study (32, 35). The changing aspects of the CTP hold greater dynamics in comparison to serum bilirubin level in various conditions, such as prediction of mortality in patients with cirrhosis. The improvement in the serum albumin levels in the present study can contribute to the increase in the total intake of proteins and the anti-catabolic effects of BCAA. In line with the preceding findings, an *in-vitro* study reports that, among three amino acids in BCAA, leucine plays a role in protein synthesis, which ultimately increases the synthesis and secretion of albumin by the hepatocytes (36, 37). BCAA has also been shown to improve albumin turnover (6) in cirrhotics, thus improving the net protein catabolism and serum albumin levels (38, 39). As a result, changes in the albumin levels can be attributed to comprehensive changes in CTP scores; nevertheless, a substantial quantity of evidence is required to validate the changes in scores. Even though biased by several aspects (40), CTP scores can predict prognosis, and its serial determinations can further improve its diagnostic accuracy in patients with cirrhosis (41–43). This finding is consistent with the findings of earlier randomized controlled trials, in which BCAA supplementation resulted in a significant improvement in the CTP score when compared with L-ABL and maltodextrins (11, 44).

In the present study, seven patients were withdrawn, either due to non-compliance or loss of follow-up in the BCAA group. However, even though the dietician provided adequate nutritional guidance, four patients withdrew from the study due to poor-palatability with the BCAA supplementation. The previous studies have reported non-compliance that was mainly attributed due to bitter taste, which leads to low palatability of supplements (35). Adverse effects, mostly gastrointestinal symptoms, have also been reported as a reason behind non-compliance, though no such reasons for non-compliance were reported in the present study. Poor medication compliance indeed affects the clinical effects. Hence, the pharmaceutical company needs to enhance the formulation to increase compliance.

However, there were some limitations of the study. First, the major problem was the large number of subjects who withdrew from the study, either because of non-compliance or lost follow-up. Therefore, there is a requirement for new and more palatable formulations to improve compliance. Second, further research for an optimal period with indication of BCAA supplementation should be conducted, taking into account hepatic inflammatory biomarkers, degree of malnutrition, and also to elucidate changes on the physical activity and nutritional intake would be needed to improve clinical outcomes.

## Conclusions

Long-term oral BCAA supplementation improved significant sarcopenic indicators, such as muscular strength, muscle function, muscle mass, and prognostic markers in patients with advanced LC, according to this study. Treatment with BCAA results in oral BCAA supplementation reduced cirrhosis-related complications, especially the development or worsening of hepatic encephalopathy, though further long-term follow-up is required to assess in-depth event-free survival. As a result, long-term oral BCAA supplementation can improve the clinical condition of patients with advanced liver disease.

## Data Availability Statement

The data generated and/or analyzed in the BCAAS study are for academic purposes and are available on appropriate requests from the corresponding author.

## Ethics Statement

The studies involving human participants were reviewed and approved by Nims University Rajasthan Jaipur Ethics Committee. The patients/participants provided their written informed consent to participate in this study.

## Author Contributions

AS: conceptualization, investigation, validation, and writing—original draft. AM: methodology and project administration. DN: conceptualization, validation, resources, writing—original draft, supervision, and funding. RR: conceptualization, writing, reviewing, editing, supervision, and funding. PS: investigation, validation, and writing—original draft. PR: investigation, formal analysis, and writing—original draft. SS: investigation, formal analysis, writing, reviewing, and editing. PM and SA: investigation, writing, reviewing, and editing. BT: conceptualization, resources, writing, reviewing, editing, supervision, and funding. All authors contributed to the article and approved the submitted version.

## Funding

Funding support for nutrition supplements (branch chain amino acid and lacto-albumin) was provided from NIMS University Rajasthan, Jaipur, India. No other funding was received from any sources. All other decisions on design, data collection, analysis and interpretation, and publication were independent of the funding sources.

## Conflict of Interest

The authors declare that the research was conducted in the absence of any commercial or financial relationships that could be construed as a potential conflict of interest.

## Publisher's Note

All claims expressed in this article are solely those of the authors and do not necessarily represent those of their affiliated organizations, or those of the publisher, the editors and the reviewers. Any product that may be evaluated in this article, or claim that may be made by its manufacturer, is not guaranteed or endorsed by the publisher.
