# Supplementary material for: Impact of Branched Chain Amino Acid on Muscle Mass, Muscle Strength, Physical Performance, Combined Survival, and Maintenance of Liver Function Changes in Laboratory and Prognostic Markers on Sarcopenic Patients With Liver Cirrhosis (BCAAS Study): A Randomized Clinical Trial
Source: Front Nutr. 2021 Sep 22;8:715795. doi: 10.3389/fnut.2021.715795 (PMC8494250; doi:10.3389/fnut.2021.715795)
Supplement: Supplementary file 1 [file Table_1.DOCX]

| **Group** | **BCAA Group** | | | | **L-ALB Group** | | | | ***p value**** |
| --- | --- | --- | --- | --- | --- | --- | --- | --- | --- |
|  | **Baseline** | **Week 24** | **Effect size** | **Δ (%)** | **Baseline** | **Week 24** | **Effect size** | **Δ (%)** |  |
| Hand grip strength | 23.79 ± 5.28 | 25.94 ± 5.14 | 0.41 | 17.64 | 24.9 ± 5.81 | 25.96 ± 5.94 | 0.15 | 0.90 | **0.02** |
| Gait speed | 0.83 ± 0.07 | 1.12 ± 0.04 | 0.50 | 3.90 | 0.86 ± 0.04 | 0.94 ± 0.13 | 0.83 | 1.63 | 0.26 |
| Total abdominal muscle area | 48.86 ± 2.50 | 50.27 ± 3.07 | 0.50 | 16.01 | 48.87 ± 1.81 | 49.72 ± 2.35 | 0.40 | 8.98 | **<0.01** |
| Fat fold tricep | 11.96 ± 1.61 | 13.37 ± 1.98 | 0.78 | 3.03 | 11.81 ± 2.19 | 12.81 ± 2.81 | 0.39 | 1.04 | **0.035** |
| Mid-arm muscle circumference | 48.86 ± 2.15 | 50.27 ± 2.47 | 0.60 | 13.78 | 48.87 ± 3.17 | 49.72 ± 3.42 | 0.25 | 6.47 | **0.015** |
| Mid-upper arm circumference | 25.28 ± 3.17 | 26.7 ± 3.48 | 0.42 | 2.67 | 26.02 ± 4.27 | 27.25 ± 4.63 | 0.27 | 1.02 | **0.047** |
| *Independent-sample t test, compared the Δ(%) between two groups.  Statistically significant at *p*<0.05 | | | | | | | | | |

**Supplementary Table 1:** Comparison of changes in sarcopenic parameters between BCAA group and L-ALB group over time of 24 weeks.
